# Transabdominal Preperitoneal Versus Lichtenstein Procedure for Inguinal Hernia Repair in Adults: A Comparative Evaluation of the Early Postoperative Pain and Outcomes

**DOI:** 10.7759/cureus.41886

**Published:** 2023-07-14

**Authors:** Victor Dumitrescu, Dragos Serban, Daniel Ovidiu Costea, Dan Dumitrescu, Florin Bobirca, Bogdan Geavlete, Dan Georgian Bratu, Laura Tribus, Crenguta Serboiu, Catalin Alius, Corneliu Tudor, Ana Maria Dascalu, Mihail Silviu Tudosie, Bogdan Serban, Doru Florian Moga

**Affiliations:** 1 Department of General Surgery, Faculty of Medicine, Carol Davila University of Medicine and Pharmacy, Bucharest, ROU; 2 Faculty of Medicine, Ovidius University of Constanța, Constanța, ROU; 3 Department of Urology, Faculty of Medicine, Carol Davila University of Medicine and Pharmacy, Bucharest, ROU; 4 Faculty of Medicine, “Lucian Blaga” University of Sibiu, Sibiu, ROU; 5 Department of Internal Medicine, Faculty of Dental Medicine, Carol Davila University of Medicine and Pharmacy, Bucharest, ROU; 6 Department of Radiology, Oncology and Hematology, Faculty of Medicine, Carol Davila University of Medicine and Pharmacy, Bucharest, ROU; 7 Department of Ophthalmology, Faculty of Medicine, University of Medicine and Pharmacy Carol Davila, Bucharest, ROU; 8 Department of Clinical Toxicology, Faculty of Medicine, Carol Davila University of Medicine and Pharmacy, Bucharest, ROU; 9 Department of Orthopaedics, Faculty of Medicine, Carol Davila University of Medicine and Pharmacy, Bucharest, ROU; 10 Faculty of Medicine, “Lucian Blaga” University of Sibiu, Bucharest, ROU

**Keywords:** miopectineal orifice (fruchaud), lichtenstein procedure, level of motor functionality, perceived level of pain, tapp, inguinal hernia

## Abstract

Inguinal hernia repairs are one of the most common procedures in general surgery. In addition to classical open surgery, laparoscopic techniques, such as transabdominal preperitoneal (TAPP) and total extraperitoneal (TEP) hernia repair, have gained acceptance and are increasingly used for inguinal hernia repairs, and these three techniques are the only standards for inguinal hernia repairs. This study aimed to compare the results of inguinal hernia repairs in adult patients using the TAPP patch technique and Lichtenstein techniques regarding the level of pain perceived one day after surgery and the number of days of hospitalization. A two-year study was performed on 129 patients who underwent TAPP vs. 109 patients who underwent Liechtenstein hernia repair. Our results revealed statistical significance for both variables (Tpain(233) = -7.12, p< 0.001, d=2.92; Tdays of hospitalization(233) = -31.34, p< 0.001, d=4.01). TAPP is a safe method for inguinal hernia repairs, allowing quick recovery and less postoperative pain than the classical Liechtenstein technique.

## Introduction

Inguinal hernia repairs are one of the most common procedures in general surgery. While a correct estimation of the annual number of hernia surgeries is difficult, previous data report up to 20 million cases of groin hernia worldwide, with inguinal repair being the most frequent [1]. This causes an important burden upon healthcare expenditures and additional economical costs related to medical leave until return to work [2,3]. Additionally, advancements in surgical techniques, including the increasing use of laparoscopic approaches, may impact the distribution of hernia repair procedures [4-6].

Most repairs are performed under general anesthesia on an inpatient basis, and there was a significant trend toward a laparoscopic repair or an open preperitoneal repair in patients with bilateral and recurrent hernias. The most commonly used laparoscopic techniques for inguinal hernia repairs are transabdominal preperitoneal (TAPP) repair and total extraperitoneal (TEP) repair [7]. Moreover, in selected cases, hernia surgery may be performed safely on an ambulatory basis [8,9].

Both classical and laparoscopic techniques are currently used, based on the availability, surgeon’s experience, and patients’ selection. The mesh use provides a tension-free repair and is associated with a lower risk of recurrences [10].

However, there is no strong evidence to support one approach to be more efficient than the other, especially in challenging situations, such as the voluminous hernial sac or Amyand hernia [11-14]. There are few randomized studies to compare open with laparoscopic techniques for inguinal hernia repairs [15-18]. Previously published meta-analyses showed comparative outcomes in terms of complications and postoperative outcomes for both surgical approaches [2,17,18]. While some evidence showed lower pain after TAPP repair, Scheuermann et al. found no significant statistical difference in a meta-analysis of eight randomized studies [18].

This study aimed to compare the outcomes of the TAPP vs Lichtenstein procedure for inguinal hernia repairs in terms of pain perceived one day after surgery measured by the Inguinal Pain Questionnaire (IPQ) score, early postoperative complications, and the number of days of hospitalization.

## Materials and methods

A two-year prospective study was carried out between July 2021 and June 2023, in the Fourth Surgery Department of the University Emergency Hospital Bucharest. Data were collected from adult patients admitted for elective hernia surgery with their freely expressed consent. The approval of the Ethics Committee of the University Emergency Hospital Bucharest, no. 2316 was obtained for this project.

Selection of the patients included in the study group

All patients underwent general clinical examination, blood tests, EKG, and imaging studies (abdominal ultrasound, CT - where applicable). The inclusion criteria in the two groups were age greater than or equal to 18 years; the general condition should be at least satisfactory; the level of motor activity should be over 40%. Exclusion criteria were co-morbid conditions making the patients unfit for general anesthesia; morbid obesity (body mass index > 30); complicated hernia in an emergency (strangulated, incarcerated hernia); suspected intra-abdominal malignancy.

Patients were included into two groups, randomly, without any particular selection criteria. All patients operated on during the study period complying with the inclusion and exclusion criteria were enrolled into two study groups: one to whom the laparoscopic transabdominal preperitoneal technique was applied (N=126) and one to whom the classical Lichtenstein method was applied (N=109) for inguinal hernia repair. Informed consent for the surgical procedure was obtained in each after a reasonable disclosure [19].

The research design is experimental with pre-and post-test testing. Both the first and second groups were tested before (T0), and after (T1) inguinal hernia surgery, and then the results were compared. All patients were followed up for one year after inguinal hernia surgery.

Pain assessment tools

We have used the following scales and applications:

Activity Assessment Scale (AAS) is a scale based on 13 motor-evaluated items of different intensity: sedentary activities, ambulatory, and work/exercise activities [20]. Each item should be graded from 1 to 5 from no difficulty in performing the respective activity to not being able to do it. Total AAS scores and subscales are transformed to produce a range of 0-100, and higher values indicate higher functional activity.

For our sample, the internal consistency index expressed by the Cronbach-α index of this scale was equal to 0.89.

The IPQ is a seven-step scale that evaluates pain perception in terms of pain behavior, with additional monitoring of pain duration. The pain in the operated groin is compared to the level of pain in the opposite one and with the worst pain experienced during the previous week [21]. For our sample, the internal consistency index expressed by the Cronbach-α index of this scale was equal to 0.84.

Statistical analysis

Statistical analysis of the data was performed using R Studio (Posit, Boston, USA), JASPER (Jaspersoft; Tibco, San Francisco, California, USA), IBM SPSS Statistics for Windows, Version 23 (Released 2015; IBM Corp., Armonk, New York, United States), and JAMOVI software (Version 2.3; Sydney, Australia; retrieved from https://www.jamovi.org). The ANOVA test was used for continuous variables. Pearson chi-square and Fisher's exact test were used to evaluate the association between discrete variables. Fisher's exact test and independent samples t-test were applied to assess whether the two groups were homogeneous in terms of general condition, type of hernia, and level of involvement in motor activities (level of motor functionality). The independent samples t-test was also used to illustrate the advantages of using the TAPP method over the Lichtenstein method for inguinal hernia repairs.

## Results

Demographic and clinical data of the patients included in the study

The study involved 235 patients aged between 21 and 90 years, who were divided into two groups: the TAPP group (126 patients) and the Lichtenstein group (109 patients). The mean age was 61.3 +/-15.61 years, with a mean of 54.17+/-16.5 years for the TAPP group and 62.3+/-14.71 years for the Lichtenstein group (p=0.012). Most of the patients were males (218; 92.7%), with no significant differences between the two study groups (118 patients; 92.1% in the TAPP group vs. 100 patients, 92.5% in the Lichtenstein group. p=0.913).

According to the European Hernia Society classification, the most common types of hernia in the study group were L2 (96 patients, 40.9%); M2 (39 patients, 16.6%), and L1 (34 patients, 14.5%).

One hundred and four patients experienced pain before inguinal hernia surgery above level 5, while 131 experienced pain below the medium level. On the other hand, only 12.3% of the participants had a motor function index below 50%, while 87.7% had a motor function index above 50%. Most of the participants had a motor function index of 52%, a sign that the inguinal hernia was causing disability (Table 1).

**Table 1:** General data of the patients included in the study ASA: American Society of Anesthesiologists; AAS: Activity Assessment Scale; IPQ: Inguinal Pain Questionnaire

Type of hernia		Number	%
	L1	34	14.5
	L2	96	40.9
	L3	3	1.3
	M1	8	3.4
	M2	39	16.6
	M3	3	1.3
	L1M1	10	4.3
	L1M2	12	5.1
	L2M1	16	6.8
	L2M2	6	2.6
	L2F	2	0.9
	M3F	6	2.6
	Total	235	100
ASA risk score			
	I	184	78.3
	II	51	21.7
gender			
	M	218	92.7
	F	17	7.3
AAS			
	45	1	0.4
	47	4	1.7
	48	10	4.3
	49	14	6
	50	11	4.7
	51	39	16.6
	52	81	34.5
	53	33	14
	54	17	7.2
	55	14	6
	56	6	2.6
	57	2	0.9
	58	2	0.9
	59	1	0.4
Preoperative 1-day IPQ			
	1	3	1.3
	2	18	7.7
	3	43	18.3
	4	67	28.5
	5	49	20.9
	6	34	14.5
	7	16	6.8
	8	4	1.7
	9	1	0.4

Homogeneity check of the two groups

To highlight the effectiveness of the TAPP method, we formed two groups out of the total of 235 patients: one to whom the TAPP method was applied (N=126) and one to whom a classical abdominal wall repair method was applied (N=109).

Tables 2, 3 show that the two groups are homogeneous in terms of general condition, type of hernia, level of motor function, and level of perceived pain before the surgery for inguinal hernia repair (p>0.05). The motor functionality level was t(233)=-2.52, p=0.06, p>0.05; F preoperative perceived pain level (1)=1.23, p=0.26, p>0.05; F type hernia(11)=2.16, p=0.16, p>0.05; F general condition (1)=1.17, p=0.21, p>0.05.

**Table 2:** T test for homogeneity among the study groups AAS: Activity Assessment Scale

	t	df	p
Preoperative AAS	-2.521	233	0.062

**Table 3:** Comparative analysis for preoperative pain perception, type of hernia, and overall condition between the two study groups

Fisher's exact test			
	χ²	df	p
F preoperative perceived pain level	1.230	1	0.267
F type hernia	21.661	11	0.167
F overall condition	1.175	1	0.212
N	235		

Perioperatively, we used routine antibiotics, and a broad-spectrum cephalosporin antibiotic (ceftriaxone) was used 30 minutes before any cutaneous incision. Furthermore, we used antibiotic therapy for patients with external drains (in most of our patients we use routinely external drains for 12-24 hours after the surgery), so the use of antibiotics is regular for all patients. As an anticoagulant medication, we used one dose of low-molecular-weight heparin (Clexane)4000UI (40mg)/0,4 ml, 12 hours after surgery and encourage early mobilization. Standard pain medication in the early preoperative period was a combination of tramadol 100mg/2ml intravenous and paracetamol intravenous (Perfalgan) 10mg/ml every eight hours first 24 hours after surgery.

Most of the patients had an uncomplicated postoperative outcome in both groups: 111 patients (88%) in the TAPP group and 90 (82.5%) in the Lichtenstein group respectively (p=0.34). The types of complications encountered and their management are presented in Table 4.

**Table 4:** Postoperative complications in the TAPP group vs. Lichtenstein group TAPP:  Transabdominal preperitoneal

Type of complication	TAPP group	Management of TAPP complication	Lichtenstein group	Management of TAPP complication
Bleeding (intraoperatory)	5	Conservatory, postoperative drainage	4	Conservatory
Retroperitoneal hematoma	2	Conservatory (selective embolization) – 1 case - reintervention -1 case	0	-
Inguinal seroma	1	Conservatory	0	-
Subcutaneous hematoma (at the trocar incision)	1	Conservatory (compressive patch, local ice)	0	-
vas deferens lesion	2	Not addressed	0	-
Reversible cutaneous femoral nerve lesion	1	Conservatory	0	-
Scrotal edema	1	Conservatory	7	Conservatory
Inguinodynia	1	Conservatory	3	Conservatory
Internal hernia	1	Reintervention	0	-
Surgical site infections	0	-	6	Conservatory

The number of complications was slightly higher in the classic vs TAPP group but not statistically significant (19 vs 11, p=0.34). Most complications in the Lichtenstein group were caused by scrotal edema (seven cases, 6.4%), surgical site infections (six cases, 5.5%), and local bleeding (3.6%), all of which could be managed by a conservatory approach. Inguinodynia was reported in three cases (2.7%) in the classic group, and only in one case (0.7%) in the TAPP group; however, the difference was not statistically significant (Chi-square test, p=0.23). Inguinodynia and testicular edema were treated with an ice pack, elevated scrotum position, and anti-inflammatory medication for 10 days. For nerve lesions, vitamin K and antalgic treatment were prescribed.

In the TAPP group, there were no surgical wound infections reported. The most frequent complications were related to bleeding, intraoperatory (3.9%), retroperitoneal hematoma (1.5%), and subcutaneous hematoma (0.7%). Two cases required reintervention under general anesthesia: one for hemostasis in a case of retroperitoneal hematoma, and another due to internal hernia. No mortality was reported in the study groups.

There were statistically significant lower postoperative hospital days and level of perceived pain (p<0.001) in the TAPP group compared to the classical inguinal hernia repair group, in terms of perceived level of pain and number of days of hospitalization: Tpain(233)= -7.12, p< 0.001, d=2.92; Tdays of hospitalization(233)= -31.34, p< 0.001, d=4.01 (Table 5).

**Table 5:** Comparative analysis of the postoperative outcomes in the two study groups

T-test for the difference between the two study groups											
		t		df		p		Cohen d		SE Cohen d	
days_of_hospitalization		-31.343		233				4.100		0.290	
IPQ_1day		-7.128		233		<0.001		2.932		0.143	

**Figure 1:**
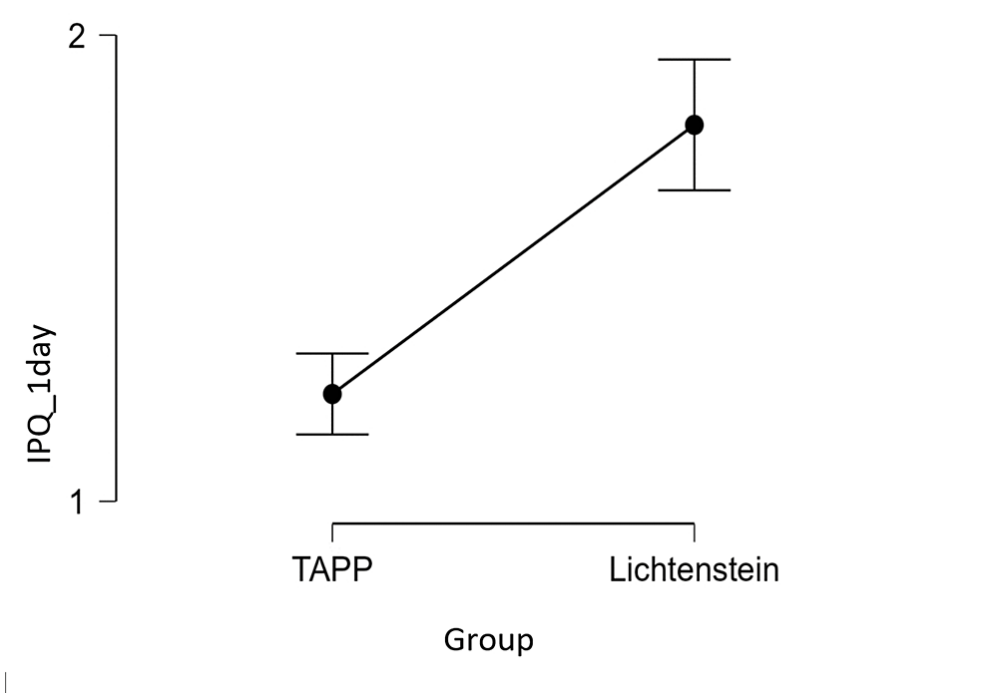
IPQ scores on day 1 after surgery in the TAPP and Lichtenstein groups TAPP:  Transabdominal preperitoneal; IPQ: Inguinal Pain Questionnaire

**Figure 2:**
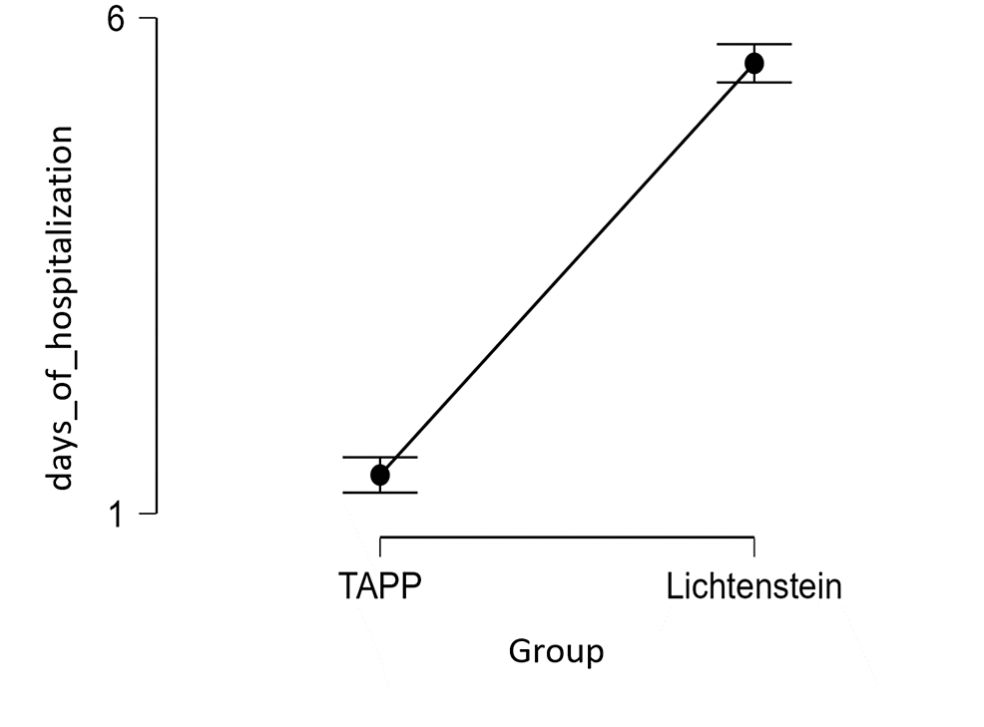
Hospital days in the TAPP group vs. Lichtenstein group TAPP:  Transabdominal preperitoneal; IPQ: Inguinal Pain Questionnaire

In our study, the TAPP method proved to be more effective in terms of the number of days of hospitalization and a low level of pain perceived by patients (M TAPP pain =1.2 and M TAPP days of hospitalization =1.3, while M Lichtenstein pain =1.8 and M Lichtenstein days of hospitalization =5.5) (Table 6 and Figures 1, 2)

**Table 6:** Comparative IPQ scores and hospital days in the two study groups TAPP:  Transabdominal preperitoneal; IPQ: Inguinal Pain Questionnaire

Descriptive statistics of the two groups													
		Group		N		M		SD		SE		Coefficient of variation	
IPQ_1day		TAPP		126		1.230		0.493		0.044		0.400	
		Lichtenstein		109		1.807		0.739		0.071		0.409	
days_of_hospitalization		TAPP		126		1.389		1.012		0.090		0.728	
		Lichtenstein		109		5.541		1.014		0.097		0.183	

## Discussion

Differences in the postoperative outcomes in TAPP vs Lichtenstein techniques were searched by various published studies in terms of healthcare expenditure, hospital days, return to work time, rate of complications, level of postoperative pain, and recurrence rate after primary repair [2,10,22,23,24].

Chronic groin pain, defined as persistent pain six months after hernia surgery, is one of the most frequent and disturbing postoperative complications, with a sign of the patient’s quality of life [22,23,25]. Its incidence varies from 6.9% to 60%, depending on the study design and methodology of data collection [25]. Previous published studies report among the risk factors for chronic groin pain high intensity preoperative and early postoperative pain, postoperative complications, neurolysis, and surgical trauma [23,25]. However, there is little previously published documentation regarding the level of early perioperative pain between TAPP and the open Lichtenstein approach, as well as the need for pain medications in the postoperative period. In the present study, we found significantly lower pain in the TAPP group, based on early postoperative evaluation on the IPQ Scale. Other studies found similar results, based on the comparison of the scores on the Visual Analogue Scale (VAS) [25-27]. One explanation is the increased tissue damage caused by open surgery. While in Lichtenstein repair, the spermatic cord and the cremaster muscle have to be dissected, during the TAPP procedure, pain is related to the dissection of the parietal peritoneum [25].

In terms of postoperative complications, we did not find significant differences in the number of postoperative complications between the two types of inguinal hernia surgery. Genital or scrotal numbness was noticed more frequently after open repair and could be correlated with an intraoperative lesion of the ilioinguinal or genitofemoral nerve during the open approach [28,29]. However, if the complications after the open Lichtenstein approach may be managed in most cases by a conservative approach, serious complications were documented after TAPP, caused by visceral or vascular injuries that need prompt reintervention [18,24].

TAPP requires access to the peritoneal cavity with the placement of a mesh through a peritoneal incision. This mesh is placed in the preperitoneal space that covers the miopectineal orifice (Fruchard). The peritoneum is then closed over the mesh leaving it between the preperitoneal tissue and the abdominal wall, where it becomes embedded by fibrous tissue [30].

However, careful closure of the peritoneum is extremely important to prevent small bowel occlusion due to preperitoneal herniation of the ileal loops [31]. Another criterion that differentiated the two procedures, TAPP and Lichtenstein, was the patient's return to work. Previous studies showed that patients returned to work sooner after TAPP repair than after Lichtenstein surgery, but the differences did not reach statistical significance [25,32].

Early recovery in terms of hospital stay and return to work was also comparatively analyzed in other previously published studies, but the results were controversial [27,29,32,33,34]. Sofi et al. [27] found that the hospitalization days were fewer with TAPP, while Hakeem and Yildiz found no significant differences [29,32].

In our study, there were also statistically significant differences in the number of hospital days between the TAPP group and the Lichtenstein group. Patients in the first group stayed in the hospital for an average of 1-2 days, while those in the second group stayed for an average of 5 and 6 days, respectively. A possible limitation of this study may be that the mean ages of the two groups compared were different, with younger patients in the TAPP group. However, there are studies in the literature that argue that age and gender do not have much of an impact as long as the surgeon uses the most appropriate methods for the patient's problems [35]. While the patients were assigned randomly to one or another study group, this is a prospective study. The lack of statistical validation for the sample size leads to possible selection bias and limits the generalizability of the results.

## Conclusions

TAPP repair showed less postoperative pain, faster discharge from the hospital, and better cosmetic results. Early postoperative complications rate was similar between the two methods. However, when appeared, the complications could be more severe following TAPP, requiring reintervention. One future direction of research would be to compare the TAPP method with another laparoscopic method. We believe that minimally invasive laparoscopic surgery can help patients to recover quickly and improve their quality of life and that the surgeon can often make a significant contribution to all this by choosing the most appropriate approach for the patient's condition.
